# Mycelium Dispersion from *Fusarium oxysporum* f. sp. *dianthi* Elicits a Reduction of Wilt Severity and Influences Phenolic Profiles of Carnation (*Dianthus caryophyllus* L.) Roots

**DOI:** 10.3390/plants10071447

**Published:** 2021-07-15

**Authors:** Janneth Santos-Rodríguez, Ericsson Coy-Barrera, Harold Duban Ardila

**Affiliations:** 1 Laboratory Research in Vegetal Metabolic Activities, Department of Chemistry, Faculty of Science, Universidad Nacional de Colombia, Ciudad Universitaria, Cra 30 No. 45-03, Bogotá 111321, Colombia; jfsantosr@unal.edu.co; 2 Bioorganic Chemistry Laboratory, Department of Chemistry, Universidad Militar Nueva Granada, Cajicá 250247, Colombia; ericsson.coy@unimilitar.edu.co

**Keywords:** carnation, *Dianthus caryophyllus*, *Fusarium oxysporum*, elicitation, biotic interaction

## Abstract

The fungal pathogen *Fusarium oxysporum* f. sp. *dianthi* (*Fod*) is the causal agent of the vascular wilt of carnation (*Dianthus caryophyllus* L.) and the most prevalent pathogen in the areas where this flower is grown. For this reason, the development of new control strategies against *Fod* in carnation has been continuously encouraged, in particular those based on the implementation of plant resistance inducers that can trigger defensive responses to reduce the disease incidence, even at lower economical and environmental cost. In the present study, the effect of the soil supplementation of a biotic elicitor (i.e., ultrasound-assisted dispersion obtained from *Fod* mycelium) on disease severity and phenolic-based profiles of roots over two carnation cultivars was evaluated. Results suggest that the tested biotic elicitor, namely, e*Fod*, substantially reduced the progress of vascular wilting in a susceptible cultivar (i.e., ‘*Mizuki*’) after two independent in vivo tests. The LC-MS-derived semi-quantitative levels of phenolic compounds in roots were also affected by e*Fod*, since particular anthranilate derivatives, conjugated benzoic acids, and glycosylated flavonols were upregulated by elicitation after 144 and 240 h post e*Fod* addition. Our findings indicate that the soil-applied e*Fod* has an effect as a resistance inducer, promoting a disease severity reduction and accumulation of particular phenolic-like compounds.

## 1. Introduction

Plant cells have the ability to continuously survey their environment and consequently activate responses associated with perceived stimuli (e.g., radiation, gravity, danger). In this way, a plant is capable of activating defense responses after recognition of signals associated with potentially dangerous events in order to achieve its survival within adverse conditions [1,2]. In general, the signal molecules that are recognized by plants, which activate responses associated with biochemical-, physiological- and/or morphological-type defense, are called elicitors [3,4]. Elicitors can be classified according to their origin into three large groups: (1) compounds of biotic origin, (2) those of abiotic origin (including those chemically synthesized), and (3) hormones [5]. Fungal chitin and bacterial flagellin are examples of some widely studied biotic elicitors. In fact, the biochemical and molecular insights of their action on different types of plant cells have already been characterized. However, there is a large number of molecules of biotic origin capable of inducing defense-associated responses in plants [6,7]. Considering the potential of such eliciting molecules in plant resistance, there is a huge interest in identifying and characterizing receptors associated with the recognition of biotic elicitors (e.g., chitin, chitosan, flagellin, oligogalacturonides, and lipopolysaccharides). In this context, elucidating the mediating mechanisms of such a response has also been pursued in order to design strategies that contribute to pathogen control, exploiting the ability of elicited plants to potentiate their self-defense response [8,9,10]. In general, the resistance-inducing capacity of these biotic-origin molecules has been the research aim in some plant species [7,11,12], since understanding the mechanisms that govern the resistance induced by the action of different elicitor substances is a crucial staring point to be efficiently implemented under field conditions [13].

Carnation is a perennial flower-producing plant commercially cultivated in various countries around the world. Exportations of this flower represented in 2020 approximately USD 205 and 250 million for Colombia and other carnation-exporting countries around the world, respectively [14]. However, this favorable carnation production is severely limited by vascular wilt, caused by the pathogen *Fusarium oxysporum* f. sp. *dianthi*. This disease has been reported in several carnation-producing countries, showing a high incidence in various commercially important cultivars ([15,16]). Therefore, the resistance induction for controlling carnation vascular wilt has attracted the recent attention of the scientific community, considering the positive environmental impact that its application would have on carnation production [17]. In other *F. oxysporum* (*Fox*)*-*hosting species, such as melon [18] and tomato [19], the application of a *Fox*-derived crude extract generated tissue sensitization and symptom reduction during a subsequent pathogen exposition. Therefore, it has been proposed that these fungal-derived extracts should contain bioactive molecules with defense-eliciting activity [18,19]. The postharvest use of this type of eliciting fraction in melon and tomato delayed wilting symptom appearance and generated an increase in phenylpropanoid pathway-related metabolite biosynthesis. Products of this pathway are well known to be associated with plant defense responses against phytopathogens [18,19]. Generally, the application of resistance inducers, either of biotic or synthetic origin, has been effectively employed to control different diseases caused by vascular wilting-related pathogens [20,21,22]. The vascular wilt-promoted resistance through biotic elicitors is particularly interesting for carnation cultivation, since international markets prefer those ornamental products not treated by synthetic agents due to the growing tendency to employ products from environmentally friendly production systems [23,24].

Plants have a diversified arsenal of specialized metabolites. It has been estimated that between 15 and 20% of their genes code for enzymes are part of specialized metabolism, and a large part of these metabolites are used as defense responses against biotic and abiotic stresses [9,25,26]. Characterization of the metabolite set associated with a phenotype has become a matter of great interest over the last 15 years, since their identification leads to a functional association through a specific role to be subsequently studied in depth. The holistic study of these molecules as a whole is known as metabolomics [27] and lets us approach the metabolome (i.e., the complete group of metabolites associated with a certain condition within a test space). In general, this can be statistically related to the cell processes occurring in the target system under particular conditions [28,29]. The metabolomics-based targeted approach has been applied in some phytopathology studies to the study of specific compound groups [30]. In this regard, for instance, the comparative analyses of phenolic-based profiles have led to delving into the roles of these compounds in different plant-pathogen interactions [31,32] and as a plant metabolic response after elicitor application [33].

Previous studies performed on carnation showed that a constitutive abundance of root-derived phenolic compounds (mostly flavonoids) is correlated with carnation resistance against vascular wilt as well as antioxidant activity [34]. In addition, the abundance of phenolic compounds was found to be higher in roots than that of the measured content in stems. Owing to roots being the first place of contact between carnation and *Fod* [35], such disease-correlated high phenolics levels can be rationalized as a defense response against this pathogen and, consequently, eliciting-promoted variations can be explored in this plant part. Therefore, the aim of the present study was to evaluate the effect of the application of a mycelium dispersion with eliciting potential, obtained from *Fod* (namely e*Fod*), on the vascular wilting severity and root phenolic-like metabolite profiles. The vascular wilting severity was evaluated for up to 8 weeks, since the symptoms associated with this disease on a carnation cuttings model can be adequately observed during this timeframe [17]. Additionally, metabolite profile variations were evaluated up to 96 h post-elicitation (hpe) in order to explore the biochemical changes that occur early, since several variations related to plant defense resistance have been reported during these timeframes in carnation [17,36,37]. In this regard, we expected that such eliciting-derived metabolic changes could be observed early, considering that a rapid defense activation would provide advantages to the host. The elicitation effect was then explored in two carnation cultivars with contrasting disease resistance levels (i.e., ‘*Mizuki*’ and ‘*Golem*’ as susceptible and resistant cultivars, respectively). The eliciting effect was accomplished through the biotic elicitor incorporation in the sowing soil. After applying the biotic elicitor treatment, the disease incidence and the average length of the carnation stems were determined. Additionally, the main phenolic compounds that were upregulated by the elicitor supplementation were statistically classified and selected from a comparative analysis of LC-MS-derived data. This information is therefore essential considering the findings of previous studies disclosing that the pre-infection levels of these metabolites may be related to disease resistance [34]. In general, the evaluation of phenolic-type metabolite changes, using targeted/untargeted metabolomics through a metabolic profiling approach, led us to point some of those upregulated phytoconstituents as a consequence of the carnation resistance induction by the biotic elicitor application.

## 2. Results

This study was divided into two stages. First, a phenotypic stage examined whether previous root elicitation with e*Fod* affected the response to the pathogen *Fod*. To do this, carnation cuttings were grown in an e*Fod*-containing substrate for six days and, subsequently, the plants were challenged with *Fod* and the severity index was measured for 8 weeks. Second, a metabolic stage was conducted in order to explore the early effect of elicitation (144 to 240 hpe) with e*Fod* on the root phenolic-based profiles.

### 2.1. Phenotypic Response of Carnation to eFod

**Figure 1:**
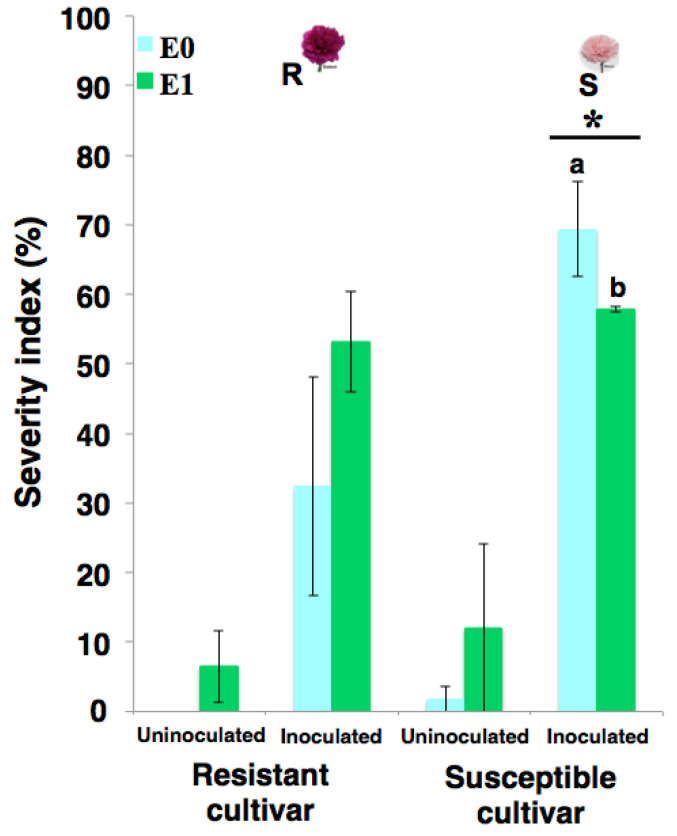
Severity index of carnation vascular wilt measured five weeks after inoculation with *Fusarium oxysporum* f. sp. *dianthi* (*Fod*) in plants previously elicited with mycelium dispersion obtained from *Fod* (e*Fod*). ‘*Mizuki*’ susceptible cultivar (S), ‘*Golem*’ resistant cultivar (R). E0: treatment without elicitor application; E1: treatments with elicitor application (1 mg/mL suspension). The variation bars represent the standard error of the mean (SEM) of three biological replicates. * Statistically-significant difference of severity index between E0 and E1 (*t*-test, *p* ≤ 0.05). Each biological replicate comprised 10 individuals. Additionally, there was no effect of the application of e*Fod* on the length of both cultivars for six weeks (Figure 2 and Figure 3).

In a first study stage, the effect of the elicitor e*Fod* on the phenotypic response of both susceptible and resistant carnation cultivars (i.e., ‘*Mizuki*’ and ‘*Golem*’, respectively) against the vascular wilting-causing pathogen was evaluated. The disease progress (e.g., symptom-appearing time and severity index) in elicited and non-elicited carnation cuttings was then monitored. This evaluation was carried out by means of two in vivo tests carried out at two different times (i.e., first trial: 20 March to 20 May 2018, second trial: 20 March to 20 May 2019). In the first trial, the monitoring of vascular wilting-related symptoms showed that, after five weeks post-*Fod* inoculation, there was a significant reduction in disease symptoms for elicited plants of the susceptible cultivar (11.5%) compared to the control group (i.e., non-elicited, inoculated plants) (Figure 1). However, the severity index difference between elicited and non-elicited plants of the resistant cultivar after *Fod* inoculation was not statistically significant, suggesting that elicitation did not have an evident effect on reducing symptoms for this resistant cultivar (Figure 1).

The behavior exhibited by the susceptible cultivar was corroborated by the results obtained after a biological replication of this experiment. This second experiment was performed using the ‘*Mizuki*’ cultivar 1 year later (Figure 4). Consistently, a positive effect of elicitation was observed by reducing the disease severity index for the elicited, inoculated plants (dark green line) compared to the non-elicited, inoculated plants (dark blue line). There was an effect on the phenotype of elicited, uninoculated plants starting at 5 wpi, showing some chlorosis in basal leaves.

### 2.2. Effect of Elicitation on the Phenolic-Based Metabolite Profiles of ‘Mizuki’ and ‘Golem’ Cultivars

In order to deepen the effect of the biotic elicitor supplementation on the carnation root biochemistry, a global comparison of the root-derived metabolic profiles was carried out. Hence, elicited and non-elicited carnation cuttings (without *Fod* inoculation) of both cultivars were only employed in this metabolic stage of the study. The aim of this exploration was oriented towards finding metabolic changes that may be related to the resistance induction. Thus, the chemical analysis was focused on phenolic-like metabolites, exploiting a simultaneous combination of mass spectrometry (MS) and ultraviolet (UV) detection through LC-DAD-ESI-MS. Owing to these compounds having a reasonable absorption at such a particular wavelength (270 nm), a selective detection was facilitated as reported in previous studies [25]. Therefore, an LC-MS-derived dataset could be filtered to have a depurated phenolic-enriched dataset. Under this approach, 195 and 211 phenolic-like features (i.e., metabolites) for the ‘*Mizuki*’ cultivar at 144 hpe and 240 hpe, respectively, were then detected and filtered, whereas the ‘*Golem*’ cultivar exhibited the presence of 200 and 199 filtered, detected features at 144 hpe and 240 hpe, respectively. Therefore, this set of features comprised the phenolic-like metabolite dataset per cultivar. These detected feature datasets were then examined through discriminant statistics to select the differential metabolites.

The variation patterns among cultivars, elicitation treatments, and post-elicitation times for root collection were initially explored using a cultivar-combined dataset through a supervised classification by partial least squares discriminant analysis (PLS-DA). Thus, the resulting scores plot (Figure 5A) clearly reflected the cultivar separation, whose principal component 1 (PC1) explained that 26.7% observed variance was dependent on the cultivar. On the other hand, the general model reflected that 15.5% variance was explained by the elicitation treatment and the post-elicitation time for root collection (i.e., 144 and 240 hpe).

In order to broaden the global comparison for exploratory purposes and pattern recognition, a second supervised analysis was carried out through orthogonal PLS-DA (OPLS-DA), using the post-elicitation time as a categorical variable for statistical supervision and dividing the whole dataset into two subgroups depending on each cultivar. The respective score plot (PC1 vs. PC2) per cultivar (Figure 5B,C), apart from confirming that the two cultivars had differentiated metabolic profiles between them, also showed that root samples collected at the different post-inoculation times (i.e., 0, 144, 156, 168, 192 and 240 hpe) were also statistically discriminated by this projection-based method. This means that the phenolic-filtered metabolic profile of roots from the two cultivars changed over time. In this sense, root samples from the ‘*Mizuki*’ cultivar, collected at early elicitation times (quadrants I and IV, Figure 5B), were separated from those collected at late times (quadrants II and III, Figure 5B). This trend was partially preserved in the ‘*Golem*’ cultivar, since later times (192 and 240 hpe) were discriminated from earlier times, including 168 hpe (Figure 5C). Owing to the observed variation for the test factors as categorical variables (i.e., cultivar and collection time), an independent comparative analysis of metabolic profiles per cultivar was justified. The former data processing was done in order to statistically select the differential metabolites, whose accumulation/production could be statistically associable to the elicitation treatment.

### 2.3. Effect of Elicitation on the Phenolic-Based Metabolite Profiles of Susceptible ‘Mizuki’ Cultivar Roots

The projection-based discriminant analysis over the dataset comprising the phenolic profiles from roots of the ‘*Mizuki*’ cultivar was also performed through PLS-DA. The resulting first two components of the model reached a 59% explained variance (Figure 6). Additionally, the performance of this supervised classification and the corresponding cross-validation (through R^2^, Q^2^, and accuracy) evidenced that the test metabolic dataset could be discriminated within a well-fitted model and, therefore, comprised a high predictability degree (Figure S1A). Actually, the cross-validation of the model performance parameters evaluated reached 90% with the first three components generated.

These facts and the respective score plot (PC1 vs. PC2, Figure 6) indicated that there was a clear group separation depending on the elicitation treatment and even the post-elicitation time (i.e., 144 and 240 hpe). In this way, this four-class comparison scheme exhibited a particular discrimination pattern along the first two PCs. The control group at 240 hpe was significantly discriminated from elicited plants at 144 hpe along PC2, indicating that these sample classes exhibited the highest metabolic variation (31.5% explained variance). Similarly, elicited plants at 240 hpe were discriminated from the control group at 144 hpe along PC1, with a close explained variance (27.5%). This projection-based discrimination trend indicated that phenolic-like profiles have temporal and eliciting-dependent variations.

Subsequently, in order to explore the metabolite-dependent behavior aiming at statistically selecting those down- and upregulated features by the elicitation effect, an analysis of variance was performed using as a dataset the semi-quantitative levels of phenolic-like compounds (normalized by sum and autoscaled) detected for the ‘*Mizuki*’ cultivar among two post-elicitation times (144 and 240 hpe). These sampling moments were selected according to the most contrasting patterns for their phenolic-based profiles recognized by the previous discriminant analysis over the time (Figure 5B,C). After this selection, 168 features were detected (multiple ANOVA, false discovery rate (FDR) ≤ 0.05). The normalized relative abundance of these detected features was used to build a heatmap that intuitively showed the global distribution of those upregulated (green boxes) and downregulated (yellow boxes) features due to the elicitation effect at one or both test post-inoculation times (Figure 7A). A Venn diagram was also constructed (Figure 7B) and supported such a global distribution on evidencing the features that had similar behavior at the different test post-elicitation times.

Upon evaluating whether elicitation had a significant effect on the mean abundance of each detected phenolic-like metabolite (multiple *t*-test, FDR ≤ 0.05), after a separated comparative analysis for each post-inoculation time, we found that 42 and 72 features were regulated at 144 and 240 hpe, respectively, in the ‘*Mizuki*’ cultivar (Figure 7B). This significant variation, due to the elicitation effect in comparison to the control group (i.e., non-elicited plants), was confirmed and validated through the analysis of receiver operating characteristic (ROC) curves, whose values of area under the curve (AUC) resulted in the maximum value (i.e., AUC = 1). Among the features discriminated as regulated, 51 features were found to be upregulated by elicitation in this susceptible cultivar. The second test eliciting time (i.e., 240 hpe) exhibited a higher number of upregulated features (i.e., 32), whereas 14 features increased specifically at 144 hpe. Only five metabolites increased significantly at both evaluated times by the effect of elicitation (Figure S2).

Detailed inspection of the mass spectral data of the phenolic-like metabolites that increased significantly by elicitation led to the respective feature annotation through identification at level 3 (i.e., putative compound type), according to the confidence levels proposed by the metabolomics standard initiative (MSI) to communicate metabolite identity by high resolution mass spectrometry (HRMS) [38]. Such annotated metabolites (Tables S1 and S2) exhibited that upregulated phenolic-like compounds involved diverse structural classes associated with different types of specialized metabolites, such as anthranilate derivatives, benzoic acids, free and conjugated flavonoids, and phenylpropanoids (i.e., metabolites containing single C_6_C_3_ building blocks) (Tables S1 and S2). The percentage distribution per post-elicitation time of these upregulated, annotated metabolites is presented in Figure 7C,D. Comparative scrutiny also shows that, at two test times, the type of phenolic compound that increased the most corresponded to glycosylated flavonoids, with 32 and 57% input for 144 and 240 hpe, respectively (Figure 7C,D, Table S1). In general, anthranilate derivatives exhibited a highly substituted anthranilate aglycone conjugated mainly with a hexose (i.e., glucose). All benzoic acids were found to be glycosylated, and most of them were methoxylated. Kaempferol- and quercetin-type flavanols predominated as the aglycone of the upregulated flavonoids.

### 2.4. Effect of Elicitation on the Phenolic-Based Metabolite Profiles of Resistant ‘Golem’ Cultivar Roots

A similar strategy to that of the ‘*Mizuki*’-derived metabolite profile comparison was used for the feature dataset of the ‘*Golem*’ cultivar. Thus, the PLS-DA-based discriminant analysis of phenolic-like metabolites of this resistant cultivar at 144 and 240 hpe delivered a 67.7% explained variance by the first two components (Figure 8).

The performance of this projection-based, cross-validated classification also indicated that the metabolite dataset fitted well for a reasonable predicting model (Figure S1B). The resulting score plot (PC1 vs. PC2, Figure 8) exhibited that sample classes were clearly separated by post-elicitation time along PC1, whereas the separation by the elicitation effect was achieved along PC2 per post-inoculation time. Evidently, the metabolic response induced by the e*Fod* application was more marked at 240 hpe than at 144 hpe, according to the observed discrimination along PC1 (54.3% explained variance) (Figure 8).

The analysis of variance performed over the dataset comprising the normalized relative abundances of those features detected in the ‘*Golem*’ roots at two evaluated times showed that 168 features were detected under the analysis conditions (multiple ANOVA, FDR ≤ 0.05). The resulting heatmap constructed with these detected features allowed the global dataset distribution to be visualized, but a low number of upregulated metabolites were visualized, involving a metabolite significantly increasing at 144 hpe, and nine metabolites at 240 hpe. Such behavior was also validated by ROC curve analysis (AUC = 1). Accordingly, these 10 statistically selected upregulated metabolites could be associated with the e*Fod* application as a metabolic response in ‘*Golem*’ roots (Figure 9A).

Their HRMS-based annotation (Tables S3 and S4) revealed that these 10 metabolites are structurally related to benzoic acids, free and conjugated flavonoids, and phenylpropanoid and polyketide derivatives (Figure 9B). Although the number of detected metabolites was similar for both cultivars, elicitation evidently promoted the accumulation/production of a higher number of shikimate/phenylpropanoid pathway-related metabolites in the susceptible cultivar than the resistant one. On the other hand, a common eliciting-induced metabolite between post-elicitation times was not observed, but the number of upregulated compounds was higher at 240 hpe, as evidenced in the susceptible cultivar (Table S4). However, the predominant metabolite class was found to be related to phenylpropanoid pathway-produced compounds. In general, the benzoic acid and polyketide derivatives were found to be glycosylated and glycosylated flavonols containing quercetin as an aglycone. In addition, free or glucose-conjugated phenylpropanoid derivatives were also evidenced.

## 3. Discussion

**Figure 2:**
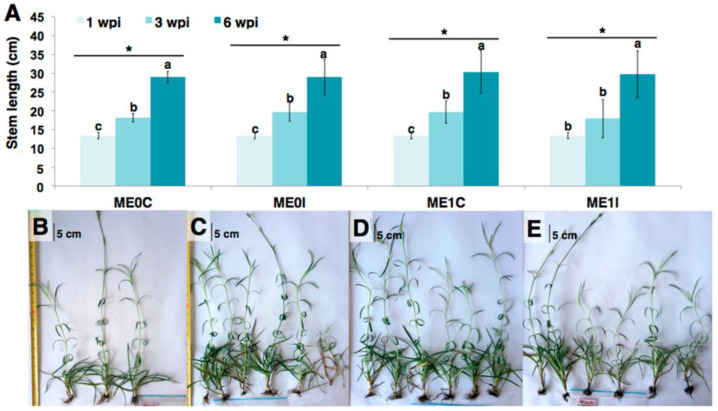
Effect of elicitation with e*Fod* and subsequent challenge with *Fod* on the stem length of cuttings of the susceptible cultivar. (**A**) Length of ‘*Mizuki*’ (M) cuttings subjected to different treatments, evaluated at three times: 1, 3, and 6 wpi (weeks post-inoculation). ME0C: non-elicited, non-inoculated cuttings; ME0I: non-elicited, inoculated cuttings; ME1C: elicited, non-inoculated cuttings. ME1I: elicited, inoculated cuttings. Photographs of ‘*Mizuki*’ cuttings at 8 wpi. (**B**) ME0C, (**C**) ME0I, (**D**) ME1C, (**E**) ME1I. The variation bars represent the standard error of the mean of three biological replicates. * Statistically-significant differences of stem lengths at each wpi per treatment (*t*-test, *p* ≤ 0.05). Three individuals were pooled for each replicate in (**B**). Six individuals were pooled for each replicate in (**C**,**D**). Scale bar—5 cm. Value for 1 wpi is the same for all treatments at the beginning of the experiment.

**Figure 3:**
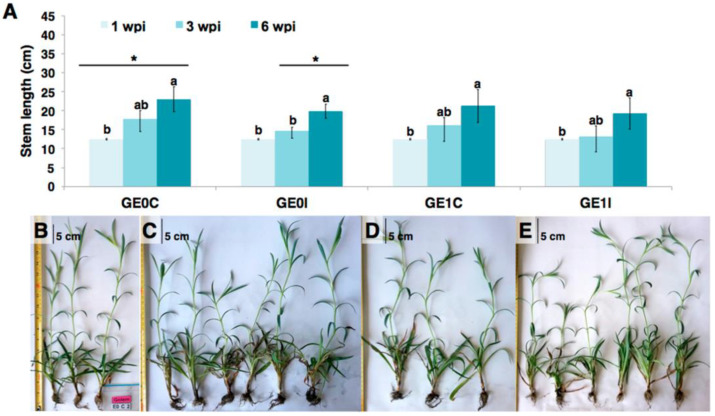
Effect of elicitation with e*Fod* and subsequent challenge with *Fod* on the stem length of cuttings of the resistant cultivar. (**A**) Length of ‘*Golem*’ (G) cuttings subjected to different treatments, evaluated at three times: 1, 3, and 6 wpi (weeks post-inoculation). GE0C: non-elicited, non-inoculated cuttings; GE0I: non-elicited, inoculated cuttings; GE1C: elicited, non-inoculated cuttings. GE1I: elicited, inoculated cuttings. Photographs of ‘*Golem*’ cuttings at 8 wpi. (**B**) GE0C, (**C**) GE0I, (**D**) GE1C, (**E**) GE1I. The variation bars represent the standard error of the mean of three biological replicates. * Statistically-significant differences of stem lengths at each wpi per treatment (*t*-test, *p* ≤ 0.05). Three individuals were pooled for each replicate in (**B**,**D**). Six individuals were pooled for each replicate in (**C**–**E**). Scale bar—5 cm. Value for 1 wpi is the same for all treatments at the beginning of the experiment.

**Figure 4:**
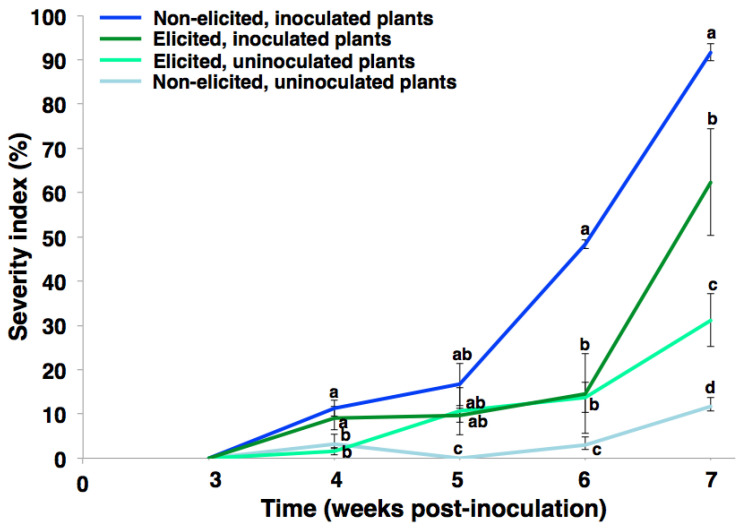
Disease severity index of vascular wilting for susceptible cultivar (‘*Mizuki*’). The variation bars represent the standard error of the mean of three biological replicates. Twenty individuals were pooled for each biological replicate. Different letters per week indicate significantly different means for each treatment according to the post-hoc Tukey test (ANOVA, *p* ≤ 0.05).

**Figure 5:**
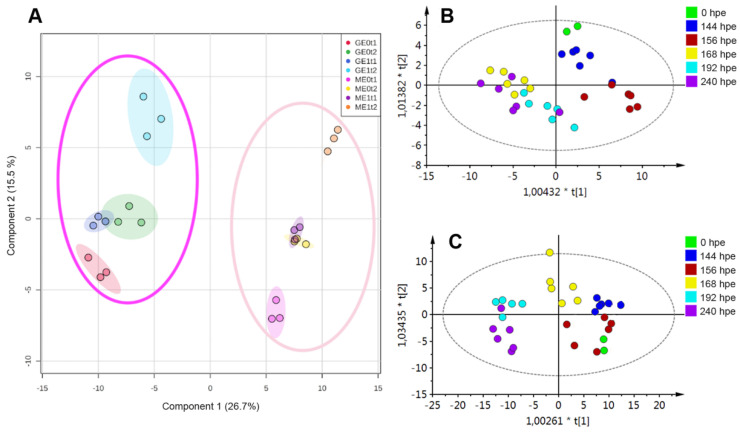
Discriminant analysis over the phenolic-like metabolite dataset after the elicitation experiment using two carnation cultivars (i.e., ‘*Mizuki*’ (M) and ‘*Golem*’ (G) as susceptible and resistant cultivars, respectively). (**A**) Partial least squares discriminant analysis (PLS-DA)-derived score plot using the entire sample set per cultivar and sampling time. Cultivar discrimination is highlighted along principal component 1 (PC1) by the dark pink ellipse (‘*Mizuki*’) and the light pink ellipse (‘*Golem*’). E0: non-elicited; E1: elicited; t1: 144 hpe; t2: 240 hpe. (**B**,**C**) Score plots created after orthogonal partial least squares discriminant analysis (OPLS-DA), using the post-elicitation times (0, 144, 156, 168, 192, and 240 h post-elicitation (hpe)) as a categorical variable for a supervised comparison of (**B**) ‘*Mizuki*’- and (**C**) ‘*Golem*’-derived profiles.

**Figure 6:**
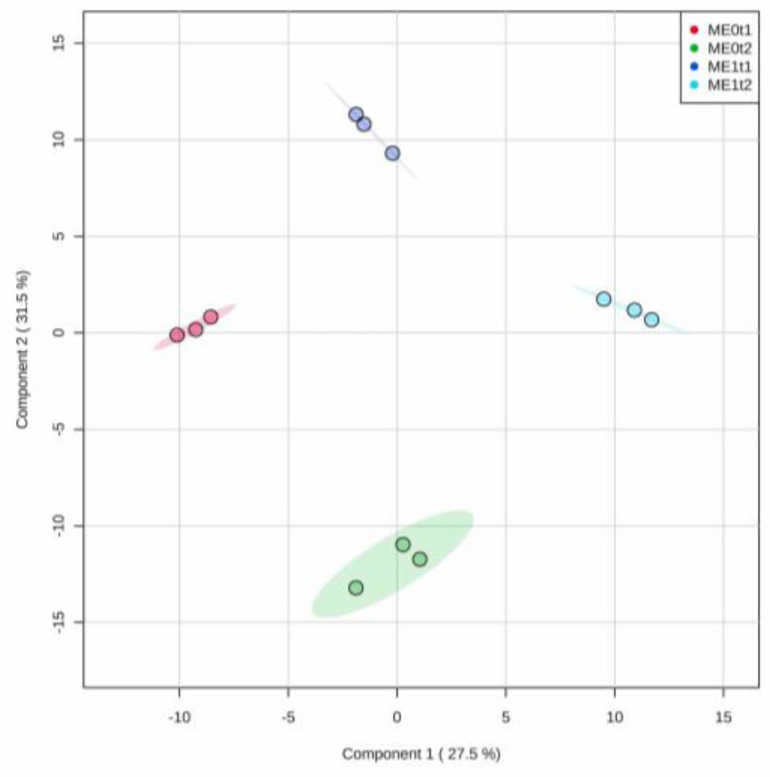
Partial least squares discriminant analysis (PLS-DA) for the phenolic-like metabolite dataset after the elicitation experiment for the ‘*Mizuki*’ (M) susceptible carnation cultivar. PC1 vs. PC2 score plot; E0: non-elicited; E1: elicited; t1: 144 hpe; t2: 240 hpe.

Resistance induction is a promising strategy for the control of parasitic-origin plant diseases [1]. In the present study, the *Fod*-obtained biotic elicitor (i.e., e*Fod*) reduced the vascular wilting severity and growth deficiency generated by the pathogen presence in carnation susceptible cultivar (i.e., ‘*Mizuki*’). The in vivo trials carried out in the present study showed for the first time that the soil application of this biotic elicitor can have a significant effect on reducing pathogenic-related disease symptoms. However, the role of e*Fod* as a resistance inducer against vascular wilting seemed to affect mostly the susceptible cultivar, since such an effect in the resistant cultivar (i.e., ‘*Golem*’) was not evidenced. This differential response is possibly related to the fact that the e*Fod* may alter some metabolic pathways already potentiated in the resistant cultivar, coinciding with the previously reported differential effect between cultivars presented by the application of this type of resistance inducer [39]. Evidence of disease symptoms in the susceptible cultivar appeared earlier during the first biological replication of the in vivo experiment to that of the second replication, possibly due to the influence of environmental factors inherent to the experiment-conducting dates. This type of observation has been previously reported in this plant model, mainly due to the environment-related impact on pathosystems with quantitative resistance [40,41,42]. However, the positive elicitation effect in carnation cuttings of the ‘*Mizuki*’ cultivar, subsequently exposed to a *Fod* challenge, was consistent after two biological replications of the in vivo experiment, corroborating that the soil application of e*Fod* induced vascular wilting resistance (Figure 1, Figure 2 and Figure 3). On the other hand, the application of a resistance inducer produced plant chlorosis, mainly at lower basal leaves, as evidenced on comparing the non-inoculated controls. These effects could be possibly due to osmotic stress and/or energetic cost. Such factors might be induced by the activation of defense response promoted by the eliciting treatment, as previously reviewed and discussed [43]. The elicitor application and the subsequent resistance induction probably had an effect on the plant physiology and, therefore, generated this lower-leaf chlorosis at the working elicitor concentration [44]. However, this high concentration achieved a significant and consistent resistance-inducing effect. This would let the chance of analyzing and detecting the determining biochemical insights of this eliciting phenomenon, which are part of the main aim of the present study, to increase. For future investigations looking for optimum parameters for its application under commercial conditions, the focus should be oriented towards selecting the suitable elicitor concentration to determine the impact on other important production-related processes.

The protection generated by the stimulation of plant tissues with an eliciting agent at high concentrations was initially described by Ross (1961) for the tobacco and tobacco mosaic virus (TMV) pathosystem [45]. During a varied number of experiments, Ross compared the size and number of lesions formed in tobacco leaves previously exposed to a TMV necrosis-generating virus with a recognized role as an immunity elicitor. Thus, after a subsequent challenge with the viral pathogen, at the opposite end of the plant, the elicited tissue presented fewer lesions and smaller sizes than those observed in control plants (i.e., non-elicited, inoculated leaves). In general, the application of different elicitors has been reported for several pathosystems [11,46], including carnation *Fod* [17]. However, the root application of biotic elicitors by direct incorporation into the sowing soil had not yet been reported. It is possible that the soil incorporation of eliciting mycelium dispersion would have an important impact on plant response due to the permanent exposure to the eliciting substances. Therefore, it is important to evaluate additional parameters in future studies such as different exposure times, stability of the e*Fod*-derived mixture into the soil, residuality, and bioavailability, among others. An extended characterization of e*Fod* is therefore required, as an eliciting-based strategy can have a lot of potential since the cultivation of this ornamental flower is currently made in hydroponic systems that require prior soil treatment by sterilization and subsequent fertilization.

In addition, the elicitor promoted variations on the total content of phenolic compounds in carnation [39]. Previous studies reported some biochemical effects associated with this type of biotic inducer [11,18,19,46]. Such biochemical variations may include the increase of phenolic-type compounds derived from the phenylpropanoid pathway. The application of a fungal-origin elicitor, obtained from the cell wall of *F. oxysporum* f. sp. *cubense* (e*Foc*), has an effect on the accumulation of phenylpropanoid-related phenols in banana roots [46]. Such a study revealed that the e*Foc* treatment induced an increase in the total phenolic content, which reached its maximum value after 16 hpe and was retained until the remaining 20 h experiment. This behavior had also been observed for the tomato *Fusarium oxysporum* f. sp. *lycopersici* (*Fol*) pathosystem. The *Fol*-derived elicitor (at 2 mg/mL) was applied to post-harvested tomato fruits and the symptom appearance was delayed for three days, and, in addition, the size of the pathogen-generating lesions decreased by 73.4% at six days after inoculation [19]. Interestingly, the application of the same elicitor from *Fol* induced phenolic compound accumulation in melon fruits and reduced the symptom severity in e*Fol*-elicited, *Fox*-inoculated melons [18]. The response observed in carnation due to the effect of e*Fod* application, as described in the previously mentioned models, might be related to an activation improvement (faster and/or stronger) defense response in the susceptible cultivar associated with the intensification of the basal resistance [12,47].

**Figure 7:**
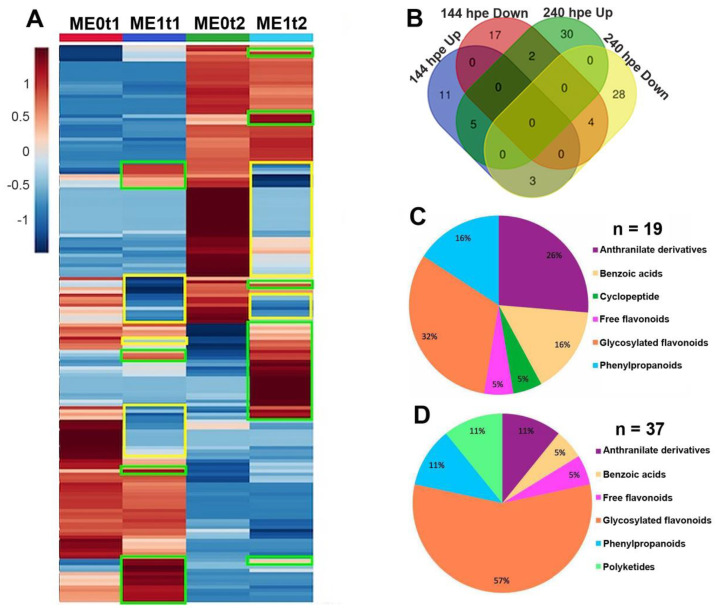
Intuitive visualization of the global distribution of down- and upregulated features (i.e., metabolites) by elicitation effect in the ‘*Mizuki*’ (M) cultivar along two post-elicitation times. (**A**) Heatmap showing the normalized relative abundance-based distribution of 168 regulated metabolites. Significantly differenced features (FDR ≤ 0.05) between elicited and non-elicited carnation cuttings are highlighted in green (upregulated) and yellow (downregulated) boxes; E0: non-elicited; E1: elicited; t1: 144 hpe; t2: 240 hpe. (**B**) Venn diagram-based classification of regulated metabolites at 144 (*n* = 19) and 240 (*n* = 37) hpe. (**C**,**D**) Classification and distribution of the annotated, upregulated metabolites by elicitation effect in the ‘*Mizuki*’ cultivar per type of specialized metabolite. (**C**) 144 hpe (*n* = 19); (**D**) 240 hpe (*n* = 37).

**Figure 8:**
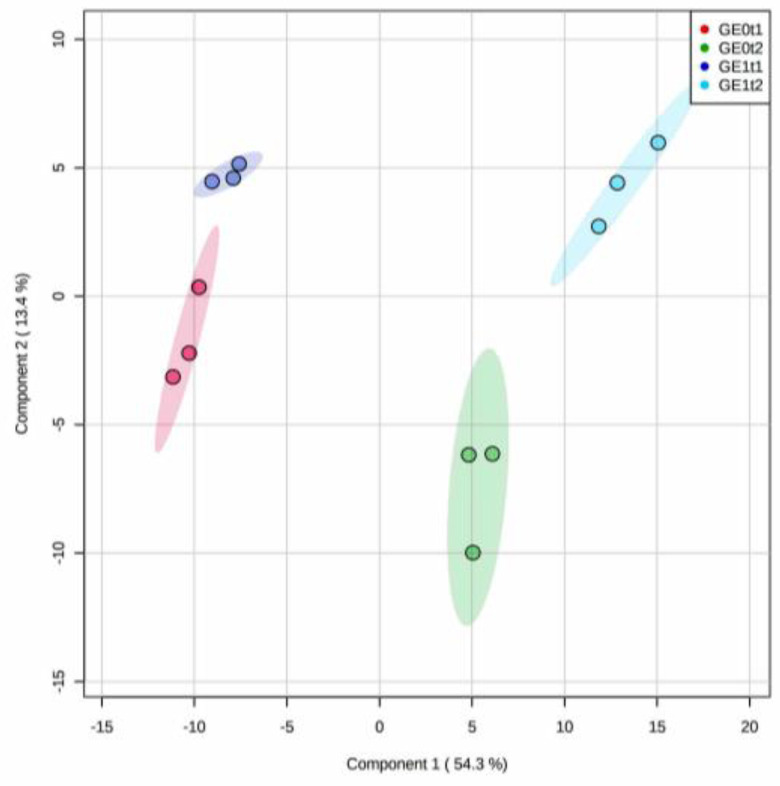
Partial least squares discriminant analysis (PLS-DA) for the phenolic-like metabolite dataset after elicitation experiment for the ‘*Golem*’ (G) resistant carnation cultivar. PC1 vs. PC2 score plot; E0: non-elicited; E1: elicited; t1: 144 hpe; t2: 240 hpe.

**Figure 9:**
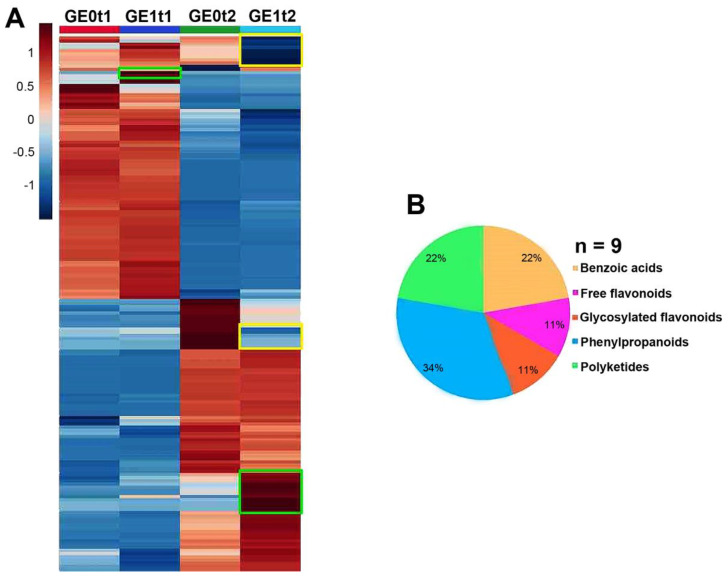
Intuitive visualization of the global distribution of down- and upregulated metabolites by elicitation effect in the ‘*Golem*’ (G) cultivar along two post-elicitation times. (**A**) Heatmap showing the normalized relative abundance-based distribution of 169 regulated metabolites. Significantly differenced metabolites (FDR ≤ 0.05) between elicited and non-elicited carnation cuttings are highlighted in green (upregulated) and yellow (downregulated) boxes; E0: non-elicited; E1: elicited; t1: 144 hpe; t2: 240 hpe. (**B**) Classification and distribution of the annotated, upregulated metabolites by elicitation effect in the ‘*Golem*’ cultivar per type of specialized metabolite at 240 hpe (*n* = 9).

A comparison of the phenolic-like metabolite profiles led to a confirmation of the accumulation of various phenolic compounds. Primarily, the changes in individual compound abundances in non-elicited carnation roots between 144 and 240 hpe were interpreted as the inherent plant physiological/biochemical differences due to the plant growth even at early times [48]. In fact, the number and abundance of particular phenolic-type metabolites were distinctly accumulated at 240 hpe in non-elicited plants of both cultivars (Figure 7A and Figure 9A), whose behavior might be part of further studies to delineate the ontogeny-related metabolite production in carnation cultivars. Regarding the elicitation effect, early studies suggested that the application of eliciting agents induced the phenolic accumulation. Such is the case of an elicitor derived from the wall of *Phytophthora parasitica*, isolated from carnation, which promoted the accumulation of a wide range of compounds derived from anthranilate (e.g., dianthramides) [49]. These compounds are typical products of carnation metabolism and have been associated with resistance against fungal-origin pathogens. In the case of carnation, a positive correlation has been reported between the increased abundance of dianthramide and diantalexin-type compounds and the *Fod* resistance in carnation stems [40]. Remarkably, in the present study, dianthramide- and diantalexin-related compounds were upregulated in roots of e*Fod*-exposed carnation cuttings of the susceptible cultivar at two post-inoculation times. However, this trend was not observed for the resistant cultivar. The dominant dianthramide-type regulated in the susceptible cultivar coincided with the type detected by Ponchet et. al., (1988) [49] in the resistant carnation cultivars previously exposed to *Fod* after 10 days post-inoculation.

On the other hand, the upregulation of glycosylated compounds was a common characteristic in the present study for elicited plants of the two carnation cultivars, as previously described [39], but involving a higher number of regulated flavonoids in the susceptible cultivar. In this context, the formation of glycosylated compounds contributes to a reactivity decrease and increases the aglycone solubility, facilitating its storage in some organelles, particularly vacuoles [50,51,52,53]. Among the annotated glycosides, quercetin and kaempferol-type flavonols were mainly found and upregulated by elicitation. Flavonols play an important role in antioxidative processes. A particular case corresponds to the dual behavior of quercetin-like compounds. Owing to their catechol moiety and when within a highly oxidizing environment, they can be oxidized through this cathecol moiety and produce antimicrobial quinones [53,54]. The increase in glycosylated compounds when exposed to e*Fod* could be interpreted as a strategy to accumulate specialized metabolites to be subsequently released at high concentrations to counteract an attack by a pathogenic agent. In this sense, the released components are oxidized compounds of these flavonols to act as antimicrobial agents [25,53]. For instance, in the particular case of onion, quercetin glycosides autoxidate after deglycosylation, generating activated oxygen (O_2_^−^), which reacts with water to produce hydrogen peroxide. This product contributes to the oxidative cleavage of quercetin and the subsequent formation of the antifungal 3,4-dihydroxybenzoic acid, among others [55]. In other cases, the presence/accumulation of these flavonols contributes to the formation of procyanidin polymers. Such polymers provide a physical–chemical barrier to limit pathogen colonization [56]. Additionally, the subsequent oxidation of flavonoids could facilitate their ability to bind to the cell wall, reinforcing the physical–chemical barrier, as well as limiting pathogen entry and its growth through the tissue. The induction of the production/accumulation of a set of multiple specialized metabolites is proposed as a strategy to generate an additive antimicrobial effect as a result of the multi-target counteracting effect against the pathogen, but also reduces the possibility of developing resistant pathogens [57].

Commonly, the accumulation of conjugated benzoic acids (CBA) can also be observed in plants under biotic interaction. For instance, CBA are not constitutively present in tobacco plants, but their accumulation is induced after a TMV infection or through biotic elicitor application [58]. However, the study by Higuera (2001) [59] demonstrated that the accumulation of a large amount of phenolic compounds (including CBA) are found constitutively in roots of resistant carnations, indicating that they may also be phytoanticipins [59]. Such results are also consistent with previously reported studies concluding that high pre-infection levels of these metabolites are related to resistance against vascular wilt caused by *Fod* in carnation [34] Among the phenolic-like metabolites upregulated by the elicitation effect, there was an increase in the abundance/accumulation of phenylpropanoid compounds (particularly caffeoyl, feruloyl, and coumaroyl types), which are precursors of the monolignols required for cell wall reinforcement [60]. On the other hand, the observed glycosylated flavonol increase, mainly in the susceptible cultivar, may suggest that the e*Fod* application primes the plant, potentiating antioxidant processes associated with the presence of kampferol- and quercetin-type compounds. The antimicrobial potential of these compounds in carnation against *Fod* has been reported in the literature, since these compounds inhibit *Fod* mycelial growth [61]. The vascular wilt-promoted resistance through biotic elicitors is particularly interesting for carnation cultivation, and unraveling the mechanisms behind its action may allow us to add this environmentally friendly strategy to the control of vascular wilt in carnation. The former aim will expand more commercial options for this flower in international markets.

## 4. Materials and Methods

### 4.1. Plant Material

Plant material used in the present study was donated by the company Florval SAS, the QFC headquarters of the Grupo Chía, located in Gachancipá town, Cundinamarca, Colombia. Pathogen-free, certified carnation cuttings (3–4 weeks of rooting) were used in all experiments. Two carnation cultivars with contrasting resistance levels to *Fod*-caused vascular wilting under field conditions were employed, namely, ‘*Mizuki*’ and ‘*Golem*’, as susceptible and resistant carnation cultivars, respectively.

### 4.2. Preparation of the Biotic-Origin Elicitor (eFod)

The eliciting mixture used in all the experiments comprised an ultrasound-assisted mycelium dispersion from the fungus *Fusarium oxysporum* f. sp. *dianthi* (*Fod*). The fungal isolate was formerly obtained from a commercial carnation crop of the company Florval SAS and molecularly characterized through genus and species following reported protocols [62,63,64]. In order to prepare the respective biotic elicitor e*Fod,* several physical treatments were performed on *Fod* mycelium to release the potentially eliciting molecules. Thus, the elicitor was prepared following the protocol described by Ardila (2007) [36] with some modifications. Briefly, 2 cm^2^ of *Fod* mycelium, previously grown for seven days on potato dextrose agar (2.5% *w*/*v*) medium (PDA), were transferred to Erlenmeyers containing liquid potato (2.5% *w*/*v*) and dextrose (1% *w*/*v*) medium (PDL). The inoculated PDL medium was incubated at 25 °C and constantly shaken (100 rpm) for seven days. Subsequently, the resulting mycelium was filtered through sterile gauze. The retained mycelium was meticulously rinsed with distilled water to remove any remaining medium, then it was sterilized in an autoclave for 15 min at 121 °C and 15 psi. The sterilized mycelium was lyophilized for 48 h and subsequently crushed under N_2_. A suitable amount of pulverized mycelium was weighed to prepare the dispersion in sterile water (1 mg/mL) to meet the total volume required for the in vivo experiments. This amount of mycelium was initially added to a portion of sterile, distilled water and the resulting dispersion was treated with ultrasound during 6 cycles of 15 s at 50 W and an amplitude of 40 each time. This sonicated dispersion was homogenized with tween-20 (0.1% *v*/*v*) and completed subsequently with sterile, distilled water to the mark of a volumetric flask. This dispersion, comprising the *Fod* mycelium-derived as e*Fod*, contained 0.053 mg total sugars/mL, following a reported procedure [65].

### 4.3. In Vivo Test: Phenotypic and Metabolic Response of Carnation by Efod Application

In order to determine the e*Fod* effect, the substrate of each tray containing test plants was supplemented with 1 mg e*Fod*/mL (200 mL), making a sufficient manually homogenization of the substrate for 60 s to get the e*Fod*-treated group (E1). On the other hand, the substrate-containing trays supplemented with sterile, distilled water were used for the control treatment, i.e., the non-treated group (E0). The substrate corresponded to a sterile mixture of silty-loamy soil and husk (1:1 ratio). Twenty-five rooted cuttings of each carnation cultivar (i.e., ‘*Golem*’ or ‘*Mizuki*’) were planted in a tray containing the e*Fod*-supplemented substrate (1 mg/mL). All trays were seeded under the same conditions for the entire period of the trial (8 weeks), involving time-controlled water irrigation, photosynthetically active radiation (80%), temperature (19 ± 7 °C), and relative humidity (70 ± 5 %). Each treatment had three biological replicates involving 10 individual plants per replicate.

#### 4.3.1. *Fod* Inoculation

In order to inoculate the cuttings, plants were required to be removed from their substrate, inoculated, and subsequently returned to their respective substrate. The delay between elicitation and inoculation was six days (144 hpe). The carnation cutting inoculation was done as previously described [58], with some modifications, by immersing the roots into a *Fod* microconidia suspension (1.0 × 10^6^ conidia/mL) for 30 s. The inoculated plants were then drained for 30 s to eliminate the conidia suspension excess and sown again in the test substrate. This substrate was previously nutrient-enriched suspension containing microconidia (1.0 × 10^3^ conidia/mL). This procedure was performed in order to favor more efficient inoculation conditions, avoiding issues related to inoculum diffusion. Similarly, the control group (non-inoculated plants) was treated under the same process, but replacing the conidia suspension with sterile, distilled water (as control root treatment) or nutrient solution without conidia (as control substrate treatment). Once the inoculation process was carried out, the cuttings were maintained with time-controlled irrigation for the entire experiment (7 weeks) under the above-described constant conditions of temperature, active photosynthetic radiation, and relative humidity.

#### 4.3.2. Evaluation of the Vascular Wilting Progress

Evaluation of the disease progress was carried out by calculating the severity index, based on the 0–4 scale previously proposed [66]. This progress was evaluated at the fifth week after *Fod* inoculation in an initial in vivo experiment. A biological experiment replicate with identical conditions, involving only the susceptible cultivar (i.e., ‘*Mizuki*’), was developed in order to corroborate the behavior observed during the first experiment replicate. The disease progress monitoring in elicited and non-elicited ‘*Mizuki*’ cuttings was determined by evaluating the severity index (SI) week by week throughout the second biological experiment replication (seven weeks). The severity index was calculated using the following equation: SI (%) = ([Σ(Class frequency × score of rating class)]/(Total number of observations) × (maximal score of rating class)) × 100 [67], where ‘score of rating class’ is related to the disease level based on the proposed scale, ‘class frequency’ is the number of plants showing that score, ‘total number of observations’ is the total number of plants within the experiment, and the ‘maximal score of rating class’ is 4.

#### 4.3.3. Preparation of Raw Metabolite-Containing Extracts

The preparation of raw extracts containing phenolic-like metabolites was carried out after root sampling at different post-elicitation times (i.e., 0, 144, 156, 168, 192, and 240 hpe). Each treatment included three biological replicates (involving roots from three individuals pooled per replicate). Hence, biological replicates (*n* = 3) were harvested, immediately rinsed with tap water, separated into roots and stems, and stored at −80 °C. Subsequently, the roots were lyophilized for 48 h and macerated under liquid nitrogen. Extraction was performed as described by Ardila [34]. The resulting mixtures were concentrated using a speedvac vacuum concentrator. Dry root extracts were reconstituted in an acetonitrile/methanol/water (1:1:12 *v*/*v*/*v*) mixture (200 µL) and this solution was subsequently filtered through a 0.2 μm polytetrafluoroethylene (PTFE) membrane, collected in an amber vial, and subsequently analyzed.

#### 4.3.4. LC-MS Analyses of Raw Extracts

Each extract was initially analyzed in order to record the metabolic profiles by reverse-phase high performance liquid chromatography (HPLC) using a Shimadzu prominence system coupled simultaneously with a multiwavelength diode array detector (DAD) SPD-M20A and a LCMS2020 mass spectrometer containing an electrospray ionization (ESI) and quadrupole analyzer (RP-HPLC-DAD-ESI-MS). The chromatographic separation was performed using a Phenomenex Luna C18 column (4.6 × 150 mm, 5 μm particle size). The mobile phase consisted of a mixture of phase A (0.1% formic acid in water) and phase B (0.1% formic acid in acetonitrile). These phases were determined during previous method standardization tests to get the optimal separation method. The gradient program was optimized as follows: 0% B (0–3 min), 0–15% B (3–20 min), 15% (20–29 min), 15–30% B (29–42 min), 30% (42–48 min), 30–100% (48–56 min), 100% (56–61 min), 100–0% (61–65 min), and 0% (65–68 min) at 1.0 mL/min flow and 30 °C column temperature. Separation monitoring was carried out at 270 nm. ESI was operated in negative ion mode (scan 100–1500 *m/z*), with the desolvation line temperature at 250 °C, nitrogen as nebulizer gas at 1.5 L/min, drying gas at 15 L/min, and detector voltage at 1.4 kV. This UV/MS-combined detection was complemented by RP-HPLC-ESI-HRMS using a Shimadzu HPLC system coupled with a Bruker MicrOToF-Q II spectrometer containing a Quadrupole-Time of Flight (QToF) analyzer and ESI. Chromatographic parameters identical to those mentioned above were implemented using a Phenomenex Luna C18 column (4.6 mm × 150 mm, 5 µm particle size). ESI was operated in positive and negative ion mode (scan 100–1500 *m/z*), with the capillary voltage at 4.5 kV, desolvation line temperature at 400 °C, nitrogen as nebulizer gas at 4 Bar, drying gas at 8 L/min, and quadrupole and collision energy at 6 and 12 eV, respectively.

### 4.4. Statistical Analyses

The severity index means of the different treatments were analyzed using an analysis of variance (ANOVA, *p*-value ≤ 0.05), with previous verification of the normal distribution of the data by Shapiro–Wilks test. A post-hoc Tukey test was subsequently used to compare multiple means and determine significant differences among treatments.

On the other hand, the statistical analysis of metabolic profiles began with the construction of the feature intensity table (FIT) as the input data matrix [observations (samples) × variables (metabolites)] [68]. Briefly, after a detailed scrutiny of the combined LC-DAD, LC-MS, and LC-HRMS data, the entire set of detected features (obtained by the commonly-used processing procedures, i.e., feature detection, deconvolution, filtering, alignment, and gap-filling) was filtered to specifically compile the phenolic-like metabolites according to the typical absorptions at 270 nm [68]. This compilation led to building the required FIT, comprising a list of filtered features (i.e., phenolic-like metabolites) for all elicited and non-elicited samples per cultivar and test post-inoculation times. Prior to the statistical analysis, data pre-processing was carried out, including (1) normalization by sum, and (2) autoscaling (scaling to unit variance) [68]. Hence, a.csv file enclosing the compiled FIT was uploaded to the web-based metabolomics tool MetaboAnalyst 5.0 [69]. Subsequently, a multiple parametric analysis (Student’s *t*-test) was applied in order to identify the features (i.e., phenolic-like metabolites) that were significantly regulated by the elicitation effect. To do so, a 95% confidence level was used (*p-*value adjusted by an FDR ≤ 0.05). The fold-change cutoff was set at 1.5 for each metabolite. Subsequently, multivariate analysis was performed for pattern recognition between samples and treatments to explain the systematic variation of the test dataset. The classification and prediction capacity of the obtained PLS-DA models were cross-validated by means of (1) performance parameters (i.e., R^2^, Q^2^, and accuracy), and (2) analysis of ROC curves (AUC = 1).

### 4.5. Annotation of Statistically Selected, Top-Ranked Metabolites by Elicitation Effect

The top-ranked features, i.e., the features exhibiting a statistically significant increase according to the *t*-test (*p-*value adjusted by an FDR ≤ 0.05) due to the elicitation effect, were annotated using the HRMS data (Tables S1 and S2). The annotation was performed following the confidence levels proposed by the MSI to communicate metabolite identity by high resolution mass spectrometry (HRMS) [38]. Thus, the molecular formula of each feature was deduced from the accurate mass measurements associated with the quasi-molecular ion [M-H]^−^ using online tool ChemCalc [70] (error ≤ 5.0 ppm). Subsequently, the feature annotation was produced after the combined diagnostic analysis of HRMS-derived data (i.e., accurate mass, molecular formula, and MS fragments), supported by phylogenetic filtering, chromatographic behavior (if possible), and data comparison with literature and different databases, such as the dictionary of natural products [71], KNApSAcK [72], and PubChem [73].

## 5. Conclusions

Eliciting-based disease control is currently an interesting strategy to be further explored and developed. To better understand the mechanism of inherent and induced resistance in carnation facing *Fod*-caused vascular wilting, the present study determined the phenotypic and metabolic responses of carnation cuttings under the presence of the *Fod*-derived elicitor (e*Fod*). The soil supplementation of e*Fod* (1 mg/mL) had a significant effect on reducing the vascular wilt progress (ca. 30% symptom decrease). This fact indicated that e*Fod* elicitation might induce resistance against this disease. From the detailed analysis of the eliciting-dependent differences of metabolic profiles, it was also evidenced that the e*Fod* supplementation had an important effect on phenolic upregulation of the two test carnation cultivars, particularly metabolites associated with the phenylpropanoid pathway. Our findings indicated that the soil-applied e*Fod* has an effect as a resistance inducer, promoting disease severity reduction and accumulation of particular phenolic-like compounds.

## Data Availability

The data that support the findings of this study are available from the corresponding author upon request.

## Supplementary Materials

The following are available online at https://www.mdpi.com/article/10.3390/plants10071447/s1, Table S1: List of metabolites induced in carnation roots (susceptible cultivar ‘*Mizuki*’) upon elicitation with e*Fod* at 144 hpe, Figure S1: Partial least squares discriminant analysis (PLS-DA) for the phenolic-like metabolite dataset after elicitation experiment. Cross-validation of parameters for the PLS-DA model performance for (A) ‘*Mizuki*’ susceptible carnation cultivar and (B) ‘*Golem*’ resistant carnation cultivar. Figure S2: Differential levels of metabolites induced in carnation roots upon elicitation with e*Fod* at both test times for the susceptible cultivar ‘*Mizuki*’. Y-axis corresponds to normalized relative abundances. Error bars correspond to standard deviation, Table S2: List of metabolites induced in carnation roots (susceptible cultivar ‘*Mizuki*’) upon elicitation with e*Fod* at 240 hpe, Table S3: List of metabolites induced in carnation roots (resistant cultivar ‘*Golem*’) upon elicitation with e*Fod* at 144 hpe, Table S4: List of metabolites induced in carnation roots (resistant cultivar ‘*Golem*’) upon elicitation with e*Fod* at 240 hpe.
