# Supplementary material for: Mycelium Dispersion from Fusarium oxysporum f. sp. dianthi Elicits a Reduction of Wilt Severity and Influences Phenolic Profiles of Carnation (Dianthus caryophyllus L.) Roots
Source: Plants (Basel). 2021 Jul 15;10(7):1447. doi: 10.3390/plants10071447 (PMC8309455; doi:10.3390/plants10071447)
Supplement: Supplementary file 1 [file plants-10-01447-s001.zip › plants-1260172-supplementary.pdf]

Journal name: Plants

SUPPORTING INFORMATION to the paper entitled: Mycelium Dispersion from *Fusarium oxysporum* f. sp. *dianthi* Elicits a Reduction of Wilt Severity and Influences Phenolic Profiles of Carnation (*Dianthus caryophyllus* L.) Roots

Janneth Santos-Rodríguez [1,2], Ericsson Coy-Barrera [2], Harold Duban Ardila [1,*]

[1] Laboratory Research in Vegetal Metabolic Activities, Department of Chemistry, Faculty of Science, Universidad Nacional de Colombia, Ciudad Universitaria, Cra 30 No. 45-03, Bogotá 111321, Colombia; jfsantosr@unal.edu.co (J.S.-R.)

[2] Bioorganic Chemistry Laboratory, Department of Chemistry, Universidad Militar Nueva Granada, Cajicá 250247, Colombia; ericsson.coy@unimilitar.edu.co (E.C.-B.)

* Correspondence: hdardilab@unal.edu.co (H.D.A); Tel.: +571-3165000 (ext. 14454); Fax: +571-3165220.

Table of contents

Table S1. List of metabolites induced in carnation roots upon elicitation with e*Fod.* Susceptible cultivar '*Mizuki*' (144 hpe).

Figure S1. Partial least squares discriminant analysis (PLS-DA) for the phenolic-like metabolite dataset after elicitation experiment. Cross-validation of parameters for the PLS-DA model performance for **(A)** '*Mizuki*' susceptible carnation cultivar and **(B)** '*Golem*' resistant carnation cultivar.

Figure S2. Differential levels of metabolites induced in carnation roots upon elicitation with e*Fod* at both test times for the susceptible cultivar '*Mizuki*'. Y axis corresponds to normalized relative abundances. Error bars correspond to standard deviation.

Table S2. List of metabolites induced in carnation roots upon elicitation with e*Fod.* Susceptible cultivar '*Mizuki*'(240 hpe).

Table S3. List of metabolites induced in carnation roots upon elicitation with e*Fod.* Resistant cultivar '*Golem*' (144 hpe).

Table S4. List of metabolites induced in carnation roots upon elicitation with e*Fod.* Resistant cultivar '*Golem*' (240 hpe).

Table S1. List of metabolites induced in carnation roots upon elicitation with e*Fod.* Susceptible cultivar '*Mizuki*' (144 hpe)

Rt (min)	*m/z* [M+H]+	*m/z* [M-H]-	Annotation (putative compound type)[a]	Type	Molecular Formula	error (ppm)	Calculated accurate mass [M-H]-	Fold Change	FDR
16.4	594.2169	592.2055	*O*-methylmethoxyanthramide-S dirhamnoside	Anthranilate derivative	$C_{31}H_{39}N_5O_7$	-4.22	592.203	2.2	0.0044
18.3	658.1989	656.1833	**dimethoxyanthramide-S diglucoside** *	Anthranilate derivative	$C_{28}H_{35}NO_{17}$	-0.91	656.1827	1.5	0.0435
20.5	450.1615	448.1229	methoxyanthramide-S glucoside	Anthranilate derivative	$C_{21}H_{23}NO_{10}$	3.35	448.1244	6.1	0.0232
38.1	346.1071	346.0917	dimethoxydianthramide-M	Anthranilate derivative	$C_{17}H_{17}NO_7$	2.89	346.0927	5.7	0.0015
45.0	520.1433	518.1296	dianthramide-S diacetylglucoside	Anthranilate derivative	$C_{24}H_{25}NO_{12}$	0.58	518.1299	5.9	0.0126
14.4	591.1359	589.1179	**methoxyphenol *O*-bis(galloyl)glucoside (isomer 1)** *	Benzoic acid	$C_{27}H_{26}O_{15}$	2.38	589.1193	2.1	0.0423
14.6	591.1337	589.1182	methoxyphenol *O*-bis(galloyl)glucoside (isomer 2)	Benzoic acid	$C_{27}H_{26}O_{15}$	1.87	589.1193	2.7	0.0435
17.1	537.1821	535.1644	diglucosyl tri-*O*-methylgallate	Benzoic acid	$C_{22}H_{32}O_{15}$	3.55	535.1663	1.9	0.0071
17.0	666.3273	664.3086	dianthin-A	Cyclopeptide	$C_{33}H_{43}N_7O_8$	1.35	664.3095	2.2	0.0423
15.4	413.1228	411.1067	(dihydroxymethoxyphenyl)fisetinidol	Flavonoid (free)	$C_{22}H_{20}O_8$	3.16	411.108	1.6	0.0212
14.9	511.1067	509.0935	*O*-methylhydroxymyricetin glucoside (isomer 1)	Flavonoid glycoside (chalcone)	$C_{22}H_{22}O_{14}$	-0.79	509.0931	1.8	0.0435
40.6	587.1115	585.1271	trihydroxyflavanone *O*-galloylglucoside (isomer 2)	Flavonoid glycoside (flavanone)	$C_{28}H_{26}O_{14}$	-4.61	585.1244	4.8	0.0054
13.4	455.1067	445.1148	***O*-methylapigenin glucoside (isomer 1)** *	Flavonoid glycoside (Flavone)	$C_{22}H_{22}O_{10}$	-3.15	445.1134	9.7	0.0212
16.9	535.1078	533.0942	kaempferol malonylglucoside	Flavonoid glycoside (Flavonol)	$C_{24}H_{22}O_{14}$	-2.06	533.0931	4.9	0.0044
19.2	525.1615	523.1428	**tri-*O*-methyldihydroquercetin glucoside** *	Flavonoid glycoside (flavonol)	$C_{24}H_{28}O_{13}$	4.59	523.1452	5.4	0.0071

44.4	493.1365	491.1182	**dihydrokaempferol acetylglucoside (isomer 1) ***	Flavonoid glycoside (flavonol)	$C_{23}H_{24}O_{12}$	1.63	491.119	5.1	0.0015
5.9	385.1147	383.0989	methoxychlorogenic acid	Phenylpropanoid	$C_{17}H_{20}O_{10}$	-2.87	383.0978	4.8	0.0054
14.2	545.1649	543.1511	di-*O*-(di-*O*-methyl)caffeoylquinic acid (isomer 1)	Phenylpropanoid	$C_{27}H_{28}O_{12}$	-1.66	543.1502	1.4	0.0435
14.3	545.1656	543.1507	di-*O*-(di-*O*-methyl)caffeoylquinic acid (isomer 2)	Phenylpropanoid	$C_{27}H_{28}O_{12}$	-0.92	543.1502	3.5	0.0051

*Metabolites induced in carnation roots upon elicitation with e*Fod* at both test times for the susceptible cultivar '*Mizuki*'.

[a]Feature annotation, through identification at level 3 (i.e., putative compound type), according to the confidence levels proposed by the metabolomics standard initiative (MSI) to communicate metabolite identity by high resolution mass spectrometry (HRMS) (Schymanski, et al. *Environ. Sci. Technol*. **2014**, *48*, 2097–2098)

Figure S1. Partial least squares discriminant analysis (PLS-DA) for the phenolic-like metabolite dataset after elicitation experiment. Cross-validation of parameters for the PLS-DA model performance for **(A)** '*Mizuki*' susceptible carnation cultivar and **(B)** '*Golem*' resistant carnation cultivar.

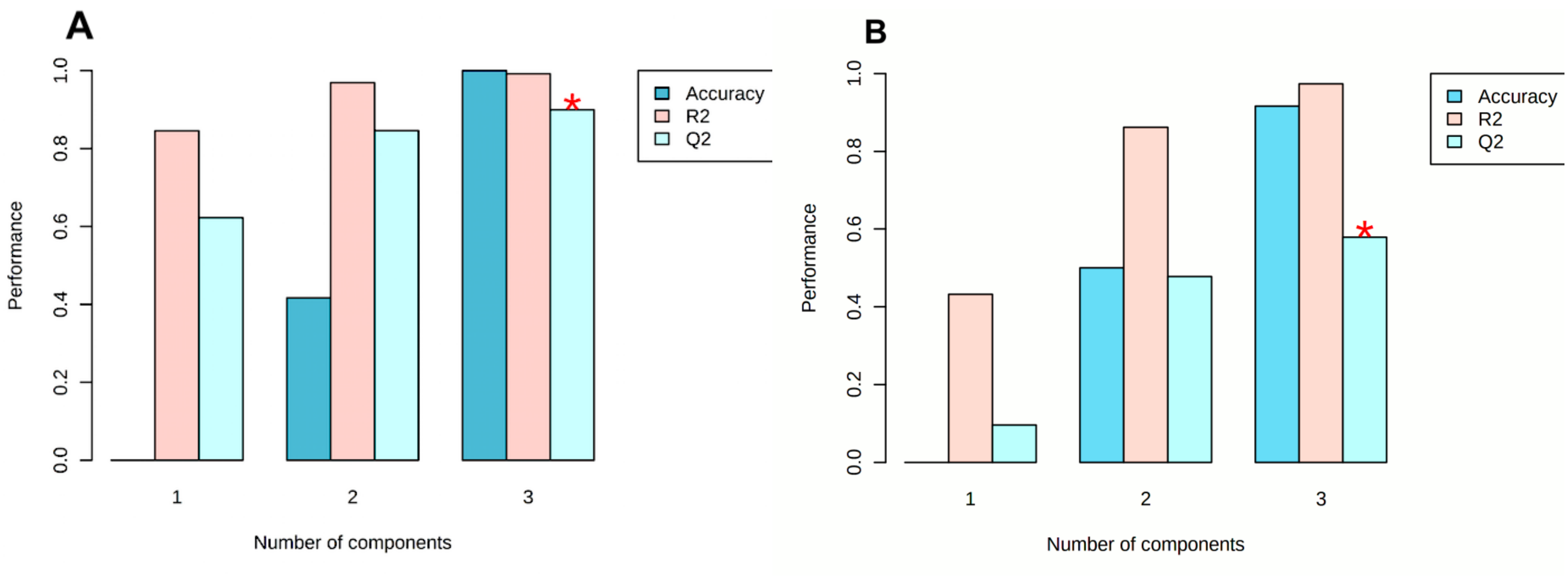

Figure S2. Levels of differential metabolites induced in carnation roots upon elicitation with e*Fod*, during both times evaluated in the susceptible cultivar '*Mizuki*'. Y axis corresponds to normalized relative abundances. Error bars correspond to standard deviation.

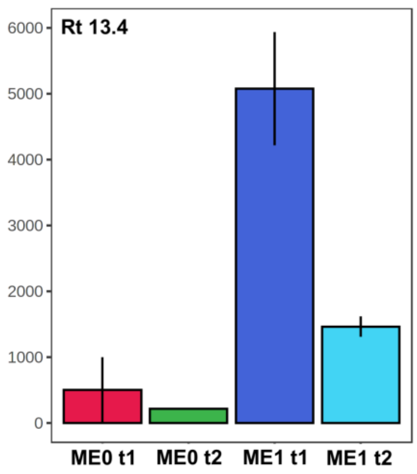

O-methylapigenin glucoside (isomer 1)

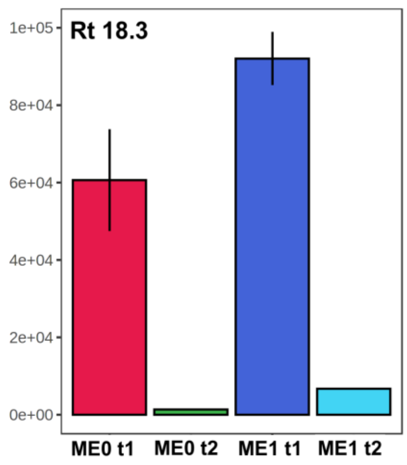

dimethoxyanthramide-S diglucoside

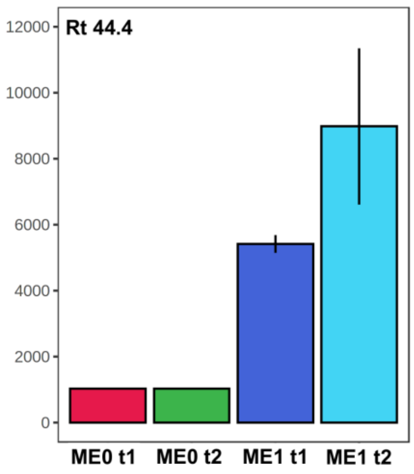

dihydrokaempferol acetylglucoside (isomer 1)

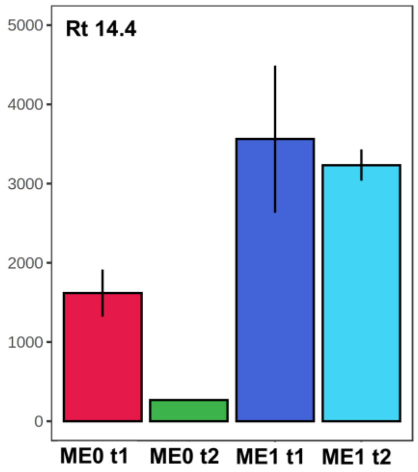

methoxyphenol *O*-bis(galloyl)glucoside (isomer 1)

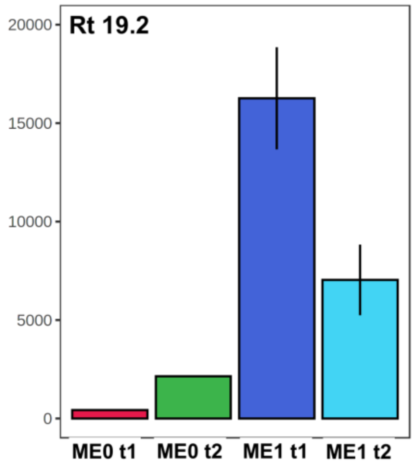

tri-*O*-methyldihydroquercetin glucoside

Table S2. List of metabolites induced in carnation roots upon elicitation with e*Fod*. Susceptible cultivar '*Mizuki*' (240 hpe)

Rt (min)	*m/z*		Annotation (putative compound type)[a]	Type	Molecular Formula	error (ppm)	Calculated accurate mass	Fold Change	FDR
	$[M+H]^+$	$[M-H]^-$					$[M-H]^-$		
18.3	658.1989	656.1833	**dimethoxyanthramide S diglucoside ***	Anthranilate derivative	$C_{28}H_{35}NO_{17}$	-0.91	656.1827	5.3	0.0001
25.7	774.2437	772.2311	anthramide-S triglucoside	Anthranilate derivative	$C_{33}H_{43}NO_{20}$	-1.42	772.23	1.4	0.0209
60.1	368.4203	366.0629	methoxyfumaroyloxydianthalexin	Anthranilate derivative	$C_{19}H_{13}NO_{7}$	-4.37	366.0613	1.6	0.0081
63.5	468.0773	466.0967	dihydroxydianthramide-S glucoside (isomer 1)	Anthranilate derivative	$C_{20}H_{21}NO_{12}$	4.08	466.0986	7.8	0.0255
14.4	591.1359	589.1179	**methoxyphenol *O*-bis(galloyl)glucoside (isomer 1) ***	Benzoic acid	$C_{27}H_{26}O_{15}$	2.38	589.1193	5.6	0.0010
43.2	611.1232	609.1079	ellagic acid rhamnosylglucoside	Benzoic acid	$C_{26}H_{26}O_{17}$	1.97	609.1091	1.6	0.0445
10.8	361.0937	359.0751	trihydroxytrimethoxyflavone (isomer 1)	Flavonoid (free)	$C_{18}H_{16}O_{8}$	4.18	359.0766	7.5	0.0255
12.6	361.0932	359.0763	trihydroxytrimethoxyflavone (isomer 2)	Flavonoid (free)	$C_{18}H_{16}O_{8}$	0.84	359.0766	2.1	0.0493
10.3	451.1266	449.1063	hydroxyisosalipurposide	Flavonoid glycoside (chalcone)	$C_{21}H_{22}O_{11}$	4.68	449.1084	2.5	0.0284
33.0	671.1957	669.1842	trihydroxyflavanone benzoylapiosylglucoside	Flavonoid glycoside (flavanone)	$C_{33}H_{34}O_{15}$	-3.44	669.1819	6.8	0.0113
34.0	635.1965	633.1807	methoxyhydroxyflavanone acetylrutinoside (isomer 1)	Flavonoid glycoside (flavanone)	$C_{30}H_{34}O_{15}$	1.90	633.1819	6.9	0.0198
39.8	587.1411	585.1269	trihydroxyflavanone *O*-galloylglucoside (isomer 1)	Flavonoid glycoside (flavanone)	$C_{28}H_{26}O_{14}$	-4.27	585.1244	1.6	0.0026
13.4	455.1067	445.1148	***O*-methylapigenin glucoside (isomer 1) ***	Flavonoid glycoside (Flavone)	$C_{22}H_{22}O_{10}$	-3.15	445.1134	6.0	0.0027
24.4	447.1279	445.1151	*O*-methylapigenin glucoside (isomer 2)	Flavonoid glycoside (flavone)	$C_{22}H_{22}O_{10}$	-3.59	445.1135	6.6	0.0113
19.2	525.1615	523.1428	**tri-*O*-methyldihydroquercetin glucoside ***	Flavonoid glycoside (flavonol)	$C_{24}H_{28}O_{13}$	4.59	523.1452	3.5	0.0318

19.8	491.1157	489.1022	kaempferol acetylglucoside	Flavonoid glycoside (flavonol)	$C_{23}H_{22}O_{12}$	2.25	489.1033	5.7	0.0020
27.4	665.2818	663.2679	hydroxymethoxyprenylflavanol diglucoside	Flavonoid glycoside (flavonol)	$C_{33}H_{44}O_{14}$	-3.92	663.2653	7.8	0.0318
29.7	649.2871	647.2723	methoxyprenylflavanol diglucoside	Flavonoid glycoside (flavonol)	$C_{33}H_{44}O_{13}$	-3.09	647.2703	5.6	0.0016
30.1	785.2511	783.2329	dimethoxyquercetin dirhanmosylglucoside	Flavonoid glycoside (flavonol)	$C_{35}H_{44}O_{20}$	2.43	783.2348	5.4	0.0001
34.8	663.1937	661.1747	kaempferol diacetylrhamnosylrhamnoside	Flavonoid glycoside (flavonol)	$C_{31}H_{34}O_{16}$	3.33	661.1769	5.9	0.0027
35.1	567.1361	565.1185	quercetin apiosilarabinoside	Flavonoid glycoside (flavonol)	$C_{25}H_{26}O_{15}$	1.42	565.1193	6.2	0.0049
35.7	503.1541	501.1378	di-*O*-methylkaempferol acetylrhamnoside	Flavonoid glycoside (flavonol)	$C_{25}H_{26}O_{11}$	3.79	501.1397	5.9	0.0016
36.4	657.1439	655.1279	*O*-methylmyricetin caffeoylglucoside (isomer 1)	Flavonoid glycoside (flavonol)	$C_{31}H_{28}O_{16}$	3.05	655.1299	2.5	0.0130
37.3	435.1285	433.1151	dihydrokaempferol rhamnoside	Flavonoid glycoside (flavonol)	$C_{21}H_{24}O_{10}$	-3.69	433.1135	5.2	0.0210
44.4	493.1365	491.1182	**dihydrokaempferol acetylglucoside (isomer 1)** *	Flavonoid glycoside (flavonol)	$C_{23}H_{24}O_{12}$	1.63	491.119	7.5	0.0198
44.7	597.1615	595.1472	kaempferol coumaroylglucoside	Flavonoid glycoside (flavonol)	$C_{30}H_{28}O_{13}$	-3.36	595.1452	5.5	0.0001
46.7	669.1834	667.1649	dihydroxyflavonol benzoylapiosylglucoside	Flavonoid glycoside (flavonol)	$C_{33}H_{32}O_{15}$	2.10	667.1663	5.5	0.0003
52.8	601.1185	599.1019	kaempferol galloylglucoside	Flavonoid glycoside (flavonol)	$C_{28}H_{24}O_{15}$	3.00	599.1037	3.7	0.0255
10.6	497.1471	495.1279	*O*-methylsilybin-B	Flavonoid-Phenylpropanoid adduct	$C_{26}H_{24}O_{10}$	2.42	495.1291	6.0	0.0026
2.6	343.1045	341.0856	caffeic acid glucoside	Phenylpropanoid	$C_{15}H_{18}O_{9}$	4.69	341.0872	1.2	0.0176
23.3	441.1051	439.0856	malonylcaffeoylquinic acid (isomer 1)	Phenylpropanoid	$C_{19}H_{20}O_{12}$	4.78	439.0877	8.1	0.0277
36.7	545.1693	543.1517	methyl di-*O*-(*O*-methyl)caffeoylquinate	Phenylpropanoid	$C_{27}H_{28}O_{12}$	-2.76	543.1502	6.4	0.0049

38.4	453.1673	451.0894	coumaroylgalloylglucoside	Phenylpropanoid	$C_{20}H_{20}O_{12}$	-3.99	451.0876	5.6	0.0010
13.0	425.1073	423.0932	pentahydroxybenzophenone glucoside	Polyketide / benzoate	$C_{19}H_{20}O_{11}$	-1.18	423.0927	1.3	0.0255
17.4	435.1253	437.1064	methoxytetrahydroxybenzophenone glucoside	Polyketide / benzoate	$C_{20}H_{22}O_{11}$	4.58	437.1084	5.6	0.0010
45.2	697.1422	695.1231	pentahydroxybenzophenone galloylcoumaroylglucoside (isomer 1)	Polyketide / benzoate	$C_{33}H_{28}O_{17}$	2.45	695.1248	6.7	0.0175
52.0	697.1416	695.1273	pentahydroxybenzophenone galloylcoumaroylglucoside (isomer 2)	Polyketide / benzoate	$C_{33}H_{28}O_{17}$	-3.60	695.1248	1.6	0.0452

*Metabolites induced in carnation roots upon elicitation with e*Fod* at both test times for the susceptible cultivar ‘*Mizuki*’.

[a]Feature annotation, through identification at level 3 (i.e., putative compound type), according to the confidence levels proposed by the metabolomics standard initiative (MSI) to communicate metabolite identity by high resolution mass spectrometry (HRMS) (Schymanski, et al. *Environ. Sci. Technol*. **2014**, *48*, 2097–2098)

Table S3. List of metabolites induced in carnation roots upon elicitation with e*Fod*. Resistant cultivar '*Golem*' (144 hpe)

Rt (min)	*m/z*		Annotation (putative compound type)[a]	Type	Molecular Formula	error (ppm)	Calculated accurate mass $[M-H]^-$	Fold Change	FDR
	$[M+H]^+$	$[M-H]^-$							
51.1	597.1618	595.1465	quercetin cinnamoylglucoside	Flavonoid glycoside (flavonol)	$C_{30}H_{28}O_{13}$	-2.18	595.1452	4.7	0.0010

[a]Feature annotation, through identification at level 3 (i.e., putative compound type), according to the confidence levels proposed by the metabolomics standard initiative (MSI) to communicate metabolite identity by high resolution mass spectrometry (HRMS) (Schymanski, et al. *Environ. Sci. Technol*. **2014**, *48*, 2097–2098).

Table S4. List of metabolites induced in carnation roots upon elicitation with e*Fod.* Resistant cultivar '*Golem*' (240 hpe)

Rt (min)	m/z		Annotation (putative compound type)[a]	Type	Molecular Formula	error (ppm)	Calculated accurate mass	Fold Change	FDR
	[M+H]+	[M-H]-					[M-H]-		
17.1	537.1821	535.1644	diglucosyl tri-O-methylgallate	Benzoic acid	$C_{22}H_{32}O_{15}$	3.55	535.1663	6.5	0.0055
48.6	327.1081	325.0917	methyl salicyloylquinate	Benzoic acid	$C_{15}H_{18}O_{8}$	1.84	325.0923	1.9	0.0451
10.8	361.0937	359.0751	trihydroxytrimethoxyflavone (isomer 1)	Flavonoid (free)	$C_{18}H_{16}O_{8}$	4.17	359.0766	2.3	0.0029
48.4	759.1974	757.1839	quercetin sambubiosylglucoside	Flavonoid glycoside (flavonol)	$C_{32}H_{38}O_{21}$	-1.58	757.1827	1.4	0.0166
2.9	283.0444	281.0288	caffeoyltartronic acid	Phenylpropanoid	$C_{12}H_{10}O_{8}$	3.20	281.0297	1.3	0.0155
11.9	369.1168	367.1032	feruloylquinic acid	Phenylpropanoid	$C_{17}H_{20}O_{9}$	-0.81	367.1029	6.5	0.0122
38.4	453.1673	451.0884	coumaroylgalloylglucoside	Phenylpropanoid	$C_{20}H_{20}O_{12}$	-1.77	451.0876	8.0	0.0451
40.2	453.1382	451.1228	dimethoxytrihydroxybenzophenone glucoside	Polyketide / benzoato	$C_{21}H_{24}O_{11}$	2.66	451.1240	5.8	0.0001
51.8	697.1412	695.1243	pentahydroxybenzophenone galloylcoumaroylglucoside (isomer 3)	Polyketide / benzoato	$C_{33}H_{28}O_{17}$	0.71	695.1248	6.1	0.0048

[a]Feature annotation, through identification at level 3 (i.e., putative compound type), according to the confidence levels proposed by the metabolomics standard initiative (MSI) to communicate metabolite identity by high resolution mass spectrometry (HRMS) (Schymanski, et al. *Environ. Sci. Technol*. **2014**, *48*, 2097–2098).
